# Evolution of temporal order in living organisms

**DOI:** 10.1186/1740-3391-3-7

**Published:** 2005-05-04

**Authors:** Dhanashree A Paranjpe, Vijay Kumar Sharma

**Affiliations:** 1 Chronobiology Laboratory, Evolutionary and Organismal Biology Unit, Jawaharlal Nehru Centre for Advanced Scientific Research, Jakkur, PO Box 6436, Bangalore 560 064, Karnataka, India

**Keywords:** circadian, adaptation, cyanobacteria, *Drosophila*, development time, lifespan

## Abstract

Circadian clocks are believed to have evolved in parallel with the geological history of the earth, and have since been fine-tuned under selection pressures imposed by cyclic factors in the environment. These clocks regulate a wide variety of behavioral and metabolic processes in many life forms. They enhance the fitness of organisms by improving their ability to efficiently anticipate periodic events in their external environments, especially periodic changes in light, temperature and humidity. Circadian clocks provide fitness advantage even to organisms living under constant conditions, such as those prevailing in the depth of oceans or in subterranean caves, perhaps by coordinating several metabolic processes in the internal milieu. Although the issue of adaptive significance of circadian rhythms has always remained central to circadian biology research, it has never been subjected to systematic and rigorous empirical validation. A few studies carried out on free-living animals under field conditions and simulated periodic and aperiodic conditions of the laboratory suggest that circadian rhythms are of adaptive value to their owners. However, most of these studies suffer from a number of drawbacks such as lack of population-level replication, lack of true controls and lack of adequate control on the genetic composition of the populations, which in many ways limits the potential insights gained from the studies. The present review is an effort to critically discuss studies that directly or indirectly touch upon the issue of adaptive significance of circadian rhythms and highlight some shortcomings that should be avoided while designing future experiments.

## Introduction

The earth's rotation around its axis causes predictable changes in the geophysical environment, thereby providing organisms with options to occupy appropriate spatio-temporal niches. Most organisms place themselves suitably in such niches using precise time-keeping mechanism(s) that can measure passage of time on an approximately 24 h scale (and hence are known as *circadian clocks*) [1]. Extensive studies over the past fifty years on a wide range of organisms have revealed some unique features of these timekeeping devices that distinguish them from other biological clocks. Some of them are summarized as follows: circadian (*circa *= approximately; *dies *= a day) clocks (i) have an inherent near-24 h periodicity, (ii) are protected from changes in temperature, nutrition and *pH*, within physiologically permissible limits, and (iii) can be tuned to oscillate with exactly 24 h period – a key property of circadian clocks known as *entrainment*, which enables living organisms to keep track of time in their local environment. These clocks are ubiquitous and are found at various levels of organization and complexity, which suggests that they must provide adaptive advantage to their owners. Circadian clocks enhance the innate ability of organisms to survive under ever-changing environments by enabling them to efficiently anticipate periodic events such as availability of food, light and mates [1-6]. It is therefore not too surprising that a wide variety of organisms such as bacteria, fungi, fish, amphibians, reptiles, insects, mammals including humans, as well as plants are able to measure time on a 24 h scale. It is believed that circadian clocks have evolved under selection pressures comprising of periodic biotic and abiotic cycles of the environment, which act on these clocks under the entrained state. As a result, precisely timed rhythmic activities confer greater adaptive advantage compared to randomly occurring activities, and in turn those clocks that enable organisms to maintain such phases (time of the day) are selected for [2,7]. Hence, the free-running phenotypes of circadian clocks are considered to be an evolutionary outcome of natural selection on entrained clocks [4]. Although circadian clocks are believed to have arisen as a result of adaptive evolution under periodic environments, there has been hardly any rigorous and conclusive empirical study to support this [2].

## Timekeeping in fluctuating environments

**Figure 1:**
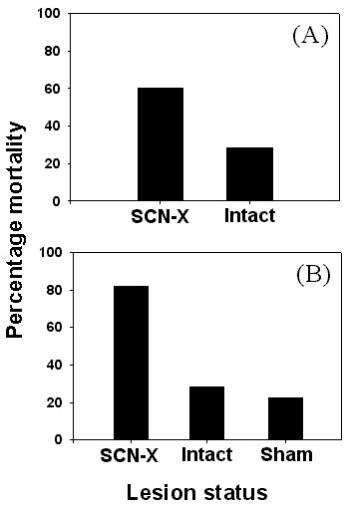
**Circadian clocks are essential for survival of organisms under natural conditions. **(A) Average survivorship of white-tailed antelope ground squirrels under semi-natural habitats. The animals were released in semi-natural habitat after surgical removal of their supra-chiasmatic nucleus (SCN) based circadian clocks. During the study period three out of five SCN-lesioned (SCN-X) individuals were predated upon as compared to two out of seven control animals. (modified after DeCoursey et al, 1997 [16]) (B) Average survivorship of free-living eastern chipmunks under natural environment. Free-living animals were captured from the study area and released back after surgical ablation of their SCN. The control animals were handled similarly and released back to the study area. During the eighty days of the study, more than 80% of SCN ablated individuals fell prey to weasels while mortality was significantly less in the surgical (sham) and intact controls. (modified after DeCoursey et al, 2000 [17])

Circadian clocks regulate a number of key behaviors in a wide variety of organisms. For example, most insects emerge as adults from their pupal case (an act known as *eclosion*) close to "dawn", when humidity is highest in the environment [8-10]. It is believed that by timing eclosion to the early hours of the day, insects prevent desiccation and thus enhance their ability to survive [11]. Circadian clocks also help organisms to restrict their activity to species-specific times of the day, which enables them to find food and mates, escape predators, and avoid undue competition between sympatric species. For example, in *Drosophila *parasitoids, activity peaks of different species occur at different times of the day, which significantly reduces intrinsic competitive disadvantage for the inferior competitor, and such temporal partitioning is achieved at least partly with the help of circadian clocks [12]. Proximal advantages of possessing circadian clocks have also been evaluated in a few studies in other animals. In a study on guillemots (*Uria lomvia*), a greater percentage of fledglings jumping out of their nests at non-species-specific times fell prey to gulls compared to those jumping during species-specific times of the day [13]. Thus, timing the jumping activity during evening hours, in synchrony with other juveniles resulted in greater chances of survival in the young ones [14]. In ground squirrels living in the wild, the hypothalamus-based circadian clock – the suprachiasmatic nucleus (SCN) – has been shown to play an important role in survival. Under laboratory conditions, SCN-ablated animals survived equally well as the controls [15], but the SCN-ablated animals quickly fell prey to feral cats when released into a semi-natural enclosure [16] (Figure 1A). This suggests that functional clocks may not be essential for survival under controlled conditions, but might become crucial under natural environments. In a subsequent marathon field study on free-living chipmunks, *Tamias striatus*, DeCoursey and coworkers [17] demonstrated that reduction in survival of the SCN-ablated animals (Figure 1B) was due to enhanced predation, perhaps due to increased nighttime restlessness.

Circadian clocks are also important for social insects such as honeybees and ants. Social insect colonies are normally faced with challenges such as changing colony sizes, time of the year, food availability, predation pressure and changing climatic conditions. The survival of these colonies under such demanding conditions requires a number of tasks to be performed simultaneously. These insects seem to have evolved division of labor, an arrangement that, in addition to enhancing efficiency of task management, promotes biological evolution of complexity and diversity [18]. In a series of experiments, Robinson and coworkers quite convincingly demonstrated that social insects use circadian clocks to efficiently manage division of labor [19]. In the colony of the Asian honeybee, *Apis mellifera*, young workers (nurses) perform tasks that can be categorized as "nursing" practically around the clock without taking any rest [20], while older honeybees (foragers) visit flowers to collect pollen and nectar in a rhythmic manner timed by well-developed circadian clocks [21]. It appears that honeybees use circadian plasticity to match age-dependent behavioural development, a phenomenon commonly known as age-polytheism [22,23]. In certain species of ants, virgin queens and males mate during nuptial flights, which occur at a species-specific time of the day, during the mating season [24,25]. Virgin queens and males use circadian clocks to time mating flight in order to encounter mating partners from neighboring colonies [24,25]. In the ant species *Camponotus compressus*, virgin queens and males use circadian clocks to time their mating flights by maintaining appropriate phase relationship with the cyclic environments, perhaps to facilitate cross-breeding between colonies and to avoid inter-species mating [26-28]. The worker castes of this species, namely the majors and media, also use circadian clocks to time their day-to-day repertoire. Foragers have well-developed circadian clocks, while soldiers guard the nest around the clock showing no obvious sign of rhythmicity. The media workers are task generalists. They are found foraging most of the time or are restricted to their colonies taking care of the queen and her brood. The activity patterns of media workers switched from nocturnal to diurnal, and clock period changed from less than 24 h to greater than 24 h, and *vice-versa*, suggesting that they are involved in shift work in their colonies. At the same time, activity of minor workers neither entrained to LD cycles nor showed any sign of free-run in DD regime, which matches well with their role as nurses. Thus, activity patterns of different castes of the ant species *C. compressus *seem to correlate well with the tasks assigned to them in their colonies [26].

Migratory birds use circadian clocks to keep track of rapidly changing day lengths in order to navigate at a specific time of the year to a more favorable climate [1]. Similarly, hibernating mammals use circadian clocks in their preparations to enter hibernation [1]. In a recent study it was reported that the Monarch butterflies, *Danaus plexippus*, undertake migratory flights every fall from northeastern America to their over-wintering grounds in central Mexico [29]. The authors demonstrated that circadian clocks play a key role in time-compensated navigation of migratory flight in Monarch butterflies [30]. European starlings use circadian clocks to compensate for changing position of the sun on long-distance journeys [31]. Similarly, golden-mantled squirrels enter hibernation in autumn when day length begins to shorten and mean daily temperature starts to drop [1]. These animals use circadian clocks to measure day lengths in order to prepare themselves for hibernation at an appropriate time of the year. On average, hibernation lasts for about 7 months with periodic wake-up bouts for sustaining brain and kidney functions through long winters. These wake-up bouts are regulated in part by circadian clocks [1], as SCN-ablation caused marked changes in the duration of wake-up bouts and the duration of hibernation [15,32]. Therefore, regularity of wake-up bouts appears to be essential even under hibernating conditions for rationing limited fat supply to last for the entire winter, as wake-up bouts are associated with muscular shivering and are metabolically expensive [1]. It is therefore evident from the above studies that circadian clocks are essential for organisms in maintaining appropriate temporal niches in their ecological and temporal environments. Previous studies suggest that circadian clocks provide proximal advantage to their owners, but they by no means serve to emphasize that these timers have any ultimate selective advantage.

## Clock fidelity

The issue of entrainment and its implications in temporal niche selection has remained central to circadian rhythm research since its inception. It is believed that natural selection acts on the phase-relationship between biological rhythm and environmental cycle, defined as the time interval between a given phase of the biological rhythm and a predictable phase of the environmental cycle. Therefore, maintenance of precise timing for behavioral and metabolic activities should be one of the most essential functions of circadian clocks, especially for organisms living in natural environments where light, temperature, humidity, food, predators and competitors fluctuate with time of the day. *How do organisms living in seemingly timeless environments such as caves, burrows and cozy apartments know the time in their local environment? *Although, no clear answer to this question exists as yet, it is believed that they do so by synchronizing their circadian clocks with the help of reliable time cues in their external environment [7,33-35]. Entrainment of circadian clocks largely depends upon two key features: phase response curve (PRC) and free-running period (τ) [7,34-36]. The free-running period is considered as an invariant property as it is assumed to remain unchanged throughout the entrainment process [35,37]. Yet, studies on a wide range of organisms have revealed that τ of circadian clocks is not an invariant property, but varies in response to different environmental conditions, often reflecting residual effects of prior environmental experience typically referred to as "after-effects" [38-42]. For example, mice exposed to LD cycles continue to exhibit rhythmic locomotor activity in DD with τ close to those of the LD cycles previously experienced, for about 100 days [33]. Such after-effects may be of some functional significance, as they could help organisms to maintain a stable phase relationship with the environmental cycles, even when environmental LD cycles are perturbed due to cloud cover or behavioural changes [33,36,43,44]. Therefore, it appears that circadian clocks have evolved a number of mechanisms to enhance their stability in ever-fluctuating environments, which in turn could increase the organism's chances of survival under natural environment [34,35].

## Dating clocks

While the proximate as well as ultimate driving forces for the evolution of circadian clocks remain largely unknown, much has been speculated as to when biological clocks might have first appeared and about what could have been the initial selection pressures that might have acted on early biological clocks [45,46]. It was believed that circadian clocks were a feature of eukaryotic organization, and that 24-h clocks would be of no advantage to prokaryotes, whose numbers double every few hours [5]. It was also believed that cellular organization of prokaryotes was too simple to accommodate complex mechanisms that are required to regulate circadian rhythms. However, it is no more a hypothesis but a fact that even primitive unicellular organisms such as cyanobacteria house functional circadian clock machinery [5]. This finding, thus pushed back the origin of circadian clocks by several hundred million years, and it is now believed that circadian clocks may have appeared on earth along with primitive life forms [46].

Circadian clocks in cyanobacteria are regulated by a cluster of three *Kai *(clock) genes – *KaiA*, *KaiB *and *KaiC *[47]. Using sequence data of these genes from several prokaryotic genomes, Dvornyk and co-workers [48] demonstrated that *Kai *genes and their homologs have quite different evolutionary histories. The *KaiC *gene is also found in Archaea and Proteobacteria [48], and among the three *Kai *genes, *KaiC *is evolutionarily the oldest. The origin of the *Kai *gene cluster appears to be one of the key events in the evolutionary history of cyanobacteria – one of the most primitive life forms on the earth. Based on the genomic data, the authors argued that circadian clocks have evolved in parallel with the geological history of earth, and natural selection, multiple lateral transfers, gene duplications and gene losses were among the major factors that further refined their evolution [48]. It is also possible that several features of circadian clocks have evolved in different organisms independently of each other and any similarity between them could be a result of convergent evolution [49].

It is also possible that the genes now involved in clock machinery formerly performed entirely different functions and were later appropriately modified to be incorporated in the clock machinery due to the changing needs of organisms in the face of cyclic selection forces. For example, *Cryptochrome *– the blue light sensitive photopigment used for circadian photoreception in *Drosophila *and plants – has been shown to exhibit striking similarity to bacterial photolyase, an enzyme involved in light-dependent DNA repair [51,52], suggesting that the *Cry *gene initially served as a key player in other cellular function(s) and might have been incorporated as part of the clock machinery at a much later stage. Regardless of the views about the original purpose of circadian clocks, there is a general belief among circadian biologists that circadian clocks evolved under the influence of cyclic factors such as light, temperature and humidity as primary selection forces. At some later stage, rhythmic activities of prey, predators and competitors might have provided additional selection pressures for its fine-tuning [46,53,54].

Several hypotheses have been put forward to explain the appearance of circadian clocks on this planet. Pittendrigh believed that circadian rhythms had evolved under selection pressure presented by environmental LD cycles, wherein photophobic processes were confined to darkness and photophilic processes to light [4]. Thus, according to Pittendrigh's "escape-from-light" hypothesis, circadian rhythms evolved to protect organisms from deleterious photo-oxidative effects of the environment by helping them reschedule light-sensitive reactions during the night [4,46,50]. For example, in cyanobacteria, key metabolic processes such as oxygen-evolving photosynthesis and oxygen-sensitive nitrogen fixation needs to be segregated in space and/or in time. Some groups of cyanobacteria have evolved special structures called heterocysts for nitrogen fixation, thus, allowing spatial segregation of the two incompatible processes, while in nonheterocyst cyanobacteria such segregation is achieved by scheduling the two processes at different times of the day [55,56]. To test the validity of the "escape from light" hypothesis, Nikaido and co-workers [57] performed experiments on unicellular alga *Chlamydomonas reinhardtii*. The survival of cells of *C. reinhardtii *was measured after exposing them to UV radiation at different times of the day. The results suggest that the cells were most sensitive to UV radiation during evening hours, when the UV component of solar radiation is normally attenuated. This suggests that the circadian timing system in *C. reinhardtii *has evolved to time crucial light-sensitive processes such as cell division during the later part of the day or in the early part of night to avoid deleterious effects of UV radiation [57].

## Conserved clocks

**Figure 2:**
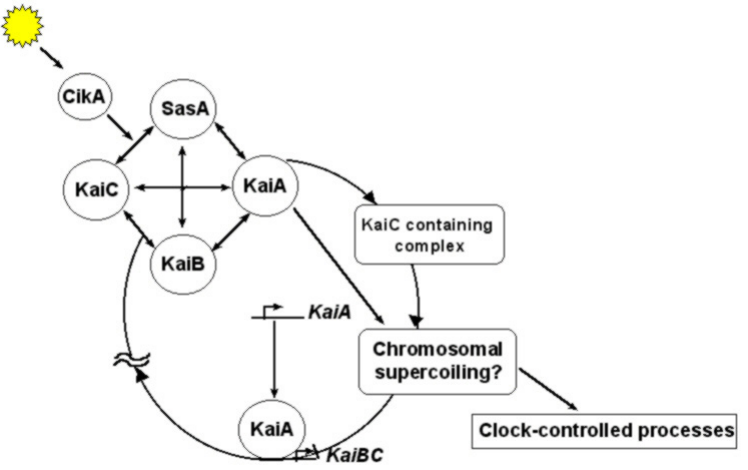
**Molecular feedback loops of cyanobacteria. **A cluster of *KaiABC *genes controls circadian rhythms in cyanobacteria. *KaiA *gene product acts as a positive regulator for *KaiBC *transcription, while *KaiBC *products along with other proteins inhibit their own transcription.

**Figure 3:**
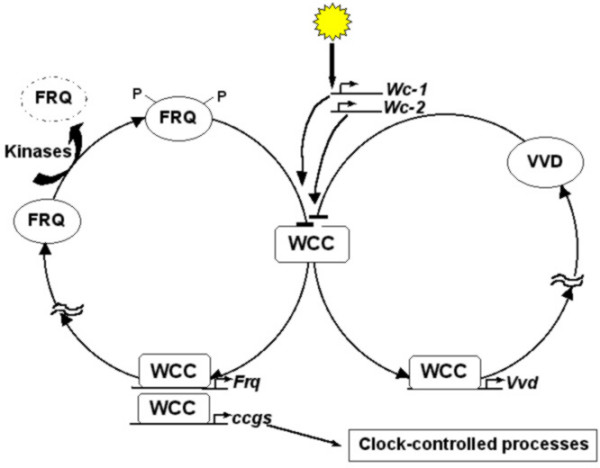
**Interlocked molecular feedback loops of *Neurospora*. **White-collar complex (WCC) acts as the transcriptional activator (positive element) of *Frequency *gene (*Frq*). The protein product of *Frq *undergoes phosphorylation in the cytoplasm under the influence of specific kinases, and subsequently acts as inhibitor of its own transcription (negative element). WCC levels are regulated by another gene called *Vivid *(*Vvd*), which in turn is regulated by WCC complex. Thus, WCC acts as one of the key components of *Neurospora *clock that connects the two loops, and hence appear to be important for the persistence of molecular oscillations. In addition, WCC is light sensitive, and appears to be crucial for light entrainment for the *Neurospora *molecular clock.

**Figure 4:**
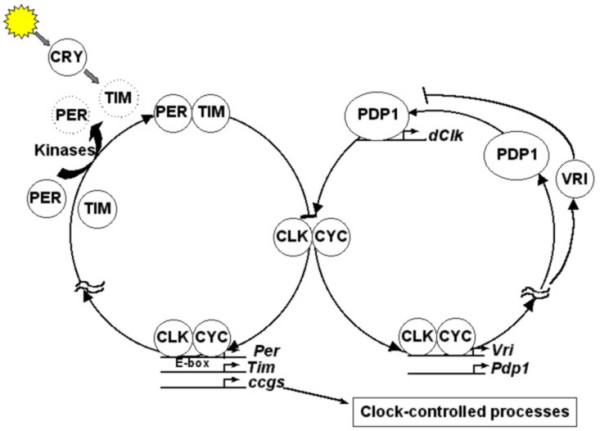
**Interlocked molecular feedback loops in *Drosophila melanogaster*. **CLOCK/CYCLE heterodimer acts as transcriptional activator (positive element) for *period *(*per*) and *timeless *(*tim*) genes. The heterodimer of PER/TIM is phosphorylated in the cytoplasm in the presence of specific kinases, and the phosphorylated complex then acts as inhibitor for its own transcription (negative element). The VRI and PDP1 proteins regulate the levels of CLK/CYC complex, which in turn are regulated by CLK/CYC. Thus, CLK/CYC heterodimer appears to be an important component that connects the two loops and is important for sustaining molecular oscillations. The protein *Cryptochrome *(CRY) has been implicated in the light entrainment pathways of the *Drosophila *molecular clock.

**Figure 5:**
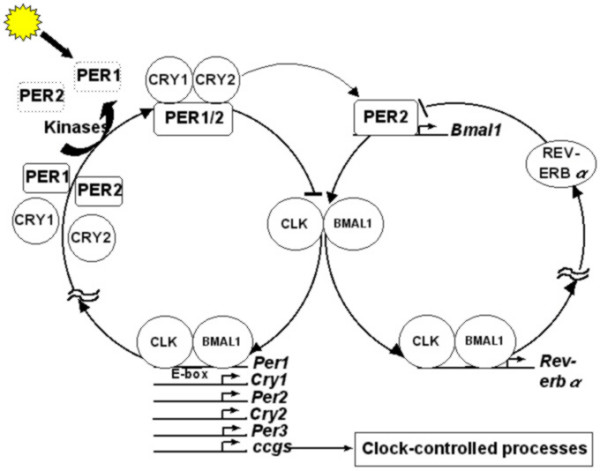
**Interlocked molecular feedback loops of mammals. **CLOCK/ BMAL1 heterodimer acts as the transcriptional activator (positive element) for *Period *(*Per*) and *Cryptochrome *(*Cry*) genes. The PER/CRY protein complex is phosphorylated in the cytoplasm by specific kinases, which then acts as inhibitor for their own transcription (negative element). In addition, these heterodimers activate *Bmal1 *transcription. CLK/BMAL1 transcription is inhibited by REV-ERBα, which in turn is regulated by CLK/BMAL1. Thus, CLK/BMAL1 heterodimer appears to be one of the key components of mammalian molecular clock, which connects the two loops. The *Period1 *gene product (PER1) is light-sensitive and appears to be important for the light entrainment of mammalian molecular clock.

Extensive genetic and molecular studies during the last three decades on model organisms such as bacteria, fungi, fruit flies and mice have provided in-depth understanding of the molecular mechanisms underlying circadian clocks. Although the finer details of the molecular players in the clock machinery appear to be different in many organisms, their functions bear remarkable degree of similarity across taxa [58] (Figures 2, 3, 4, 5). The underlying molecular mechanisms involve multiple feedback loops comprising of genes whose transcripts and/or protein products oscillate with near 24 h periodicity [59-61]. The positive elements in the molecular clockwork are transcriptional activators of one or more clock genes with DNA binding bHLH (basic Helix-Loop-Helix) motifs. These activators enhance the transcription of clock genes by binding to specific E-box sequences in the promotor region of the clock genes. This results in abundance of transcripts, which then translate to yield clock proteins. The protein products form heterodimers by interacting via a PAS (PER-ARNT-SIM) domain, and are subsequently phosphorylated in the cytoplasm by specific kinases, after which they enter the nucleus. Some heterodimers act as negative elements in the feedback loops, as their binding brings about conformational changes in the protein structures of the transcriptional activators, in a manner that they can no longer bind to the promoter region of the clock genes, thereby inhibiting their transcription. The positive elements of the loop also activate the transcription of a few clock-controlled genes (ccgs), which control overt rhythmicity directly or indirectly through yet unknown mechanisms. The molecular feedback loops are interconnected such that the protein heterodimer acting as transcriptional activator in one loop can inhibit transcription of clock genes in the other loop. Such components of molecular loops, which play dual roles in the core clock mechanisms, are particularly important for self-sustained molecular oscillations. The DNA binding bHLH domain [59,62] and the protein-protein interacting PAS domain are highly conserved in organisms ranging from cyanobacteria to mammals [63]. The *KaiA *protein in cyanobacteria [47] (Figure 2), WCC in *Neurospora *(Figure 3), CLK/CYC in *Drosophila *(Figure 4), and CLOCK/BMAL1 in mouse (Figure 5) act as transcriptional activators, of which all except *KaiA *are heterodimeric transcriptional activators. The negative elements such as *KaiC *protein in *Synechococcus*, FRQ in *Neurospora*, PER/ TIM in *Drosophila *and PER1, PER2, CRY1, CRY2 in mouse block their own transcription by interacting with transcriptional activators. In addition, WCC in *Neurospora*, CLK/CYC in *Drosophila *and CLK/ BMAL1 in mouse are some of the key elements that play dual roles; as transcriptional activators in one loop and transcriptional inhibitors in the other (Figures 2, 3, 4, 5). In addition, in many organisms genes involved in light input pathways are also involved in the core clock mechanisms [51]. The basic function of the molecular clock bears remarkable similarity in a wide range of organisms. Besides high degree of functional similarity between the molecular clocks, there is also a considerable degree of structural similarity between the clock genes of insects and mammals. The *clock *(*clk*) and *doubletime *(*dbt*) genes in *Drosophila *and mammals have considerable sequence homologies and have similar functional roles in the respective organisms [64,65]. Homologs of *per *gene have been reported in several species of *Drosophila *[66], and a number of other insect species such as housefly (*Musca domestica*) [67] and honeybee (*Apis mellifera*) [22]. Orthologs of *per *have been identified in mammalian system and more recently in Zebrafish [68]. Furthermore, the photopigment *cryptochrome *involved in the light input pathways of circadian clocks in fruit flies has been found to have remarkable structural and functional similarities to those of mammals and plants [51].

Although the overall molecular mechanisms underlying circadian clocks in various organisms are to a great extent conserved, there are also subtle differences. For example, in *Drosophila*, mRNA and protein levels of *cycle *(*cyc*), which forms an important part of the transcriptional activator CLK/CYC, do not oscillate, whereas, *dClk *mRNA as well as protein levels show robust oscillations [60]. In mammals, the level of CLK protein does not oscillate, but BMAL1 is prominently rhythmic [60]. Furthermore, in *Drosophila*, CRY acts as a photopigment and as an important component of the core clock mechanisms in the peripheral clocks [69]. In the mammalian circadian timing system, CRY is only a part of the core molecular mechanisms; the possibility of its role in light perception has been ruled out [58]. Further, in contrast to the *Drosophila *molecular clock, which consists of only one *cry *and one *per *gene, the mammalian molecular clock consists of two *Cry *genes (*mCry1 *and *mCry2*) and three *Per *genes (*mPer1*, *mPer2 *and *mPer3*) [58]. Molecular mechanisms regulating circadian clocks in *Chlamydomonas reinhardtii *have been reported to be entirely different. Extensive search for potential homologs to genes that are known to encode components of the circadian clock in other organisms has revealed that there are no obvious homologs in the *C. reinhardtii *genome, except for the kinases and phosphatases that are involved in the molecular clockwork [70]. The kinases and phosphatases in fungi, plants, flies, mammals and *C. reinhardtii *are highly conserved, and it appears that they play a key role in the clock mechanisms. One of the two CRY proteins found in *C. reinhardtii *is closely related to plant CRYs, while the other one is more similar to animal CRYs. Since there are no homologs of any known clock genes in *C. reinhardtii*, it is possible that the green alga might host novel clock mechanisms involving some novel core clock components [70]. Barring a few exceptions such as those in *Chlamydomonas *circadian timing system, the overall molecular mechanisms underlying light input pathways, rhythm generating core mechanisms and rhythm transduction mechanisms that send rhythmic signals to efferent organs bear striking structural and functional similarities in organisms ranging from cyanobacteria to mammals. Given that the behavioural and metabolic processes regulated by circadian clocks are so diverse, it is astonishing that the underlying molecular mechanisms giving rise to these varieties of rhythmic phenomena are so similar across a wide range of taxa.

## Clock for all seasons

Organisms living in temperate regions are exposed to drastic changes in photoperiod and temperature. Circadian clocks are believed to play an important role under such demanding situations [71]. Studies on several strains of *D. littoralis *originating from a wide range of geographic locations at different latitudes revealed a mild latitudinal trend in the phase and period of eclosion rhythm [72]. The northern strains had shorter period and earlier phase of eclosion compared to the southern strains [72]. Similar latitudinal clines for phase and amplitude of eclosion rhythm were also reported in *D. auraria *[71]. Since amplitude of circadian rhythm responds to changes in photoperiod as well as temperature, it was concluded that these insects use circadian clocks to sense seasonal changes in their environment [73]. Furthermore, fifty-seven European populations of *D. littoralis *showed latitudinal cline for adult diapause, where the northern populations responded to longer critical day lengths than the southern populations [72]. Later, in a separate study, clinal patterns in threonine-glycine (Thr-Gly) repeats were reported at the *period *locus in European and north African strains of *D. melanogaster *[74] and *D. simulans *[75]. The northern strains showed higher frequency of (Thr-Gly)_17 _compared to the southern strains, while the frequency of (Thr-Gly)_20 _was higher in the southern strains than in northern strains [74,75]. Further studies on the locomotor activity rhythm in these populations at 18°C and 29°C revealed that circadian clocks of the (Thr-Gly)_20 _variants had the most efficient temperature compensation ability, while this was not the case for the (Thr-Gly)_17 _variants, as they showed period shortening at lower temperatures [76]. Since clinal variation in phase and period in these strains appear to have arisen as a result of natural selection, presence of such latitudinal clines can be taken as an indirect evidence for the adaptive evolution of circadian clocks [71,72].

## Clocks for birth and death

The assumption that circadian clocks influence fitness traits has formed the basis of several studies aimed at addressing adaptive significance of circadian rhythms. It is generally believed that faster clocks speed up development and cause reduction in lifespan, while slower clocks slow down development and lengthen lifespan [77-80]. Several studies have been carried out in a variety of organisms to investigate possible links between circadian clocks and life history traits such as pre-adult development time and adult lifespan. In an extensive study on the *per *mutants of *D. melanogaster*, which display circadian rhythms with widely different periods, pre-adult development time was measured under continuous dim light (LL), very bright continuous light (VLL), continuous darkness (DD), light/dark (LD) cycles of 12:12 h, and LD 12:12 h superimposed with temperature cycles (LD 12:12 T). Under all light regimes, development time of *per *mutants was positively correlated with τ of their circadian clocks, i.e. *per*^S ^mutants (τ = 19 h) developed faster than wild type flies (τ = 24 h), which in turn developed faster than the *per*^*L *^mutants (τ = 28 h) [77]. A positive correlation between development time and clock period was seen even in absence of the overt rhythmicity under LL regime and also under entrained conditions such as those prevailing under LD cycles, which suggests that the *per *mutation has pleiotropic effects on circadian phenotype and pre-adult development time. In a recent study in *D. melanogaster*, pre-designed to bypass such pleiotropic effects, clock period and developmental time were positively correlated (faster eclosion rhythm was associated with faster development and slower oscillations accompanied slower development), thus suggesting a possible role of the periodicity of LD cycles and/or of eclosion rhythm in determining the duration of pre-adult development [80]. In a separate study on the melon fly (*Bactrocera cucurbitae*) that involved selection for faster and slower pre-adult development, faster developing lines had faster circadian clocks, whereas slower developing lines had slower circadian clocks [81]. The timing of behaviors such as locomotion and preening was shifted significantly to earlier hours of the day in faster developing lines compared to the slower developing lines. The mating peaks in the faster developing lines occurred close to dusk while most of the flies from the slower developing lines mated during the night [82]. The period of locomotor activity rhythm was shorter (τ ~ 22.6 h) in faster developing lines and longer (τ ~ 30.9 h) in the slower developing lines [83]. Although, most studies suggest a role of circadian clocks in timing pre-adult development, the robustness of such conclusion is limited by the fact that association between development time and circadian clocks in some of the studies shows very little effect of light regime, suggesting pleiotropic effects of the *per *mutation.

In a study on the *tau *mutant hamsters, heterozygous (τ ~ 22 h) animals under laboratory LD (14:10 h) cycles lived shorter than the wild type animals (τ ~ 24 h), but the average lifespan of homozygous animals (τ ~ 20 h) did not differ from those of the wild type animals [78]. Contradictory results were obtained in a similar study performed under constant dark (DD) conditions, wherein homozygous animals lived significantly longer than the wild type controls, while the average lifespan of heterozygote animals did not differ from those of the wild type controls [79]. Such differences in outcome could be due to the fact that the two studies were performed under different environmental conditions, and environmental factors are known to modify the outcome of such studies [77,78,84]. In a separate study in fruit flies (*Drosophila melanogaster*), significance of circadian clocks in physiological well being has been investigated in some detail. The lifespan of *per*^T ^(short period mutant, τ = 16 h), and *per*^L ^(long period mutant, τ = 29 h) mutants was reduced considerably compared to *per*^+ ^(wild type, τ = 24 h) flies, even when flies were maintained under LD cycles with periodicity closer to the endogenous periodicity of the mutant lines [85]. The studies discussed above serve to emphasize that lifespan of *D. melanogaster *is not regulated by the clock period; rather it is determined by the genotype of the flies, which suggests pleiotropic effect of *per *mutation on clock period and lifespan. The role of circadian clocks in determining life-history traits is likely to be important for the adaptive evolution of organisms, especially under periodic environments. Evidence at hand provides at least strongly suggestive, if not conclusive, evidence that circadian clocks control key life-history traits. They also raise a possibility that some evolutionary response of life-history traits to forces of natural selection may be partly mediated through changes in circadian clocks.

## Clocks for reproduction

The role of circadian clocks in reproductive output of *D. melanogaster *was investigated in great depth in clock mutants. Studies on loss of function mutants of *D. melanogaster *such as *per*^*0*^, *tim*^*0*^, *cyc*^*0*^, *Clk*^*jrk *^revealed that single mating among clock-deficient phenotypes result in ~ 40% lesser progeny compared to the wild type flies [86]. In general, null mutants laid fewer eggs, out of which only a few were fertile [86]. Further experiments on *per*^*0 *^and *tim*^*0 *^flies showed that the amount of sperm released from the testes to seminal vesicles of males was significantly reduced in the null mutants compared to the wild type flies [86].

Although egg-laying is rhythmic in flies of a wide range of genotypes, transcripts of *per*, and protein levels of *per *and *tim *do not oscillate in the ovaries of *Drosophila *females [87]. A constitutively high level of PER and TIM proteins were found in the follicle cells of developing oocytes throughout the day. Previous studies have demonstrated that PER and TIM interact in these follicle cells but do not translocate into the nucleus, thus leaving clock mechanisms truncated [88]. Therefore, for a long time it remained unclear as to what is the functional role of the two clock genes *per *and *tim *in the fly ovary. In a recent study, Beaver and co-workers [88] quite convincingly demonstrated that lack of functional *per *and *tim *in virgin females resulted in significantly fewer mature oocytes in *per*^*0 *^and *tim*^*0 *^flies compared to the wild type flies. Rescue of clock function in *per*^*0 *^mutants by ectopically expressing *per *in the lateral neurons alone did not enhance the production of mature oocytes [88]. Thus, suggesting that *per *and *tim *may have non-clock functions in the ovaries [88]. Fitness components and circadian phenotypes are both multigenic traits, and the underlying genes may have pleiotropic effects. Therefore, it is fair to speculate that mutations affecting clock may simultaneously reduce reproductive fitness via mechanisms that are independent of clock-related processes. Alternatively, manipulations in certain genes or processes closely related to fitness traits may also alter clock phenotype, through clock independent processes, thus leaving the issues related to adaptive significance of circadian clocks via reproductive output unresolved.

## Resonating clocks

**Figure 6:**
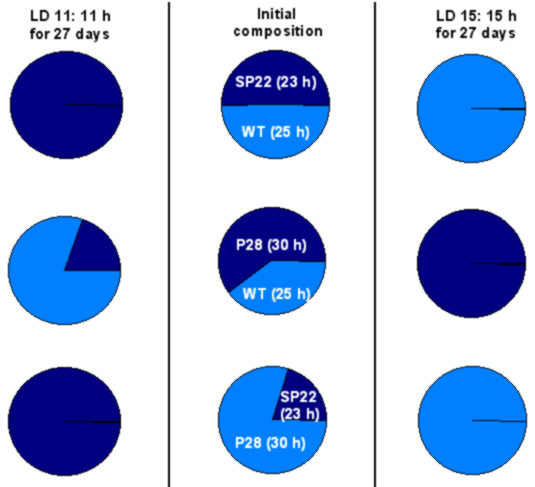
**Circadian resonance in cyanobacteria. **Rhythmic strains having different free-running periods were competed under LD cycles of different lengths. Strains whose free-running period matched that of LD cycles out-competed those with deviant periods. Middle panels represent initial composition of the competing strains. Values in the parenthesis indicate the free-running period of the cyanobacterial strains. (Figure modified after Ouyang et al, 1998 [6])

**Figure 7:**
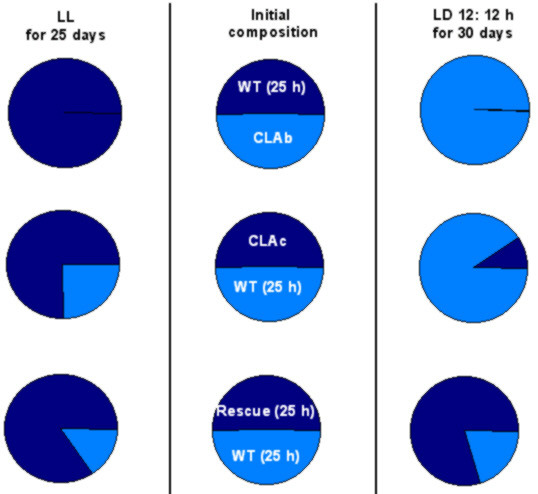
**Competition between rhythmic and arrhythmic strains of cyanobacteria. **Mutant strains with arrhythmic (CLAc), or dampened (CLAb) bioluminescence rhythm, as well as the rescued strains were competed against wild type strain under periodic and constant environments (LD cycles and LL, respectively). Rhythmic strains out competed the wild type strain under LD cycles, but the arrhythmic strains out competed rhythmic strains under LL. Middle panels represent initial composition of the competing strains. Values in the parenthesis indicate the free-running period of the cyanobacterial strains. (Figure modified after Woelfle et al, 2004 [97])

The ubiquitous presence of circadian clocks in a wide variety of phenomena and organisms suggests that they confer adaptive advantage to their owners, perhaps by enabling the organisms to synchronize to LD cycles, and thereby to maintain appropriate phase relationships between their external and internal cycles. Based on this logic, it was speculated that at the advent of early life forms on this planet several temporal patterns were present in living organisms, but only those which matched environmental periodicity managed to survive. Motivated by this thought, Pittendrigh and Bruce [89] proposed a hypothesis known as the "circadian resonance hypothesis", which states that organisms with clocks having periodicities matching those of cyclic environment perform "better" compared to those whose periodicities do not match the period of the environmental cycles. If circadian resonance were the driving force behind the evolution of circadian clocks, one would expect organisms with near-24 h periodicity to have greater fitness advantage under a 24 h environment than any other periodic or aperiodic environment. Indeed, fruit flies (*D. melanogaster*) lived significantly longer under 24 h LD cycles than either in 21 h (LD 10.5:10.5 h), 27 h (LD 13 5:13.5 h) LD cycles or under LL [90]. In blowflies (*Phormia terranovae*), lifespan of flies that were reared under 24 h LD cycles, was significantly greater under 24 h LD cycles than under any other non-24 h LD environment [91]. In a separate study on the *per *mutants of *D. melanogaster*, lifespan of male *per*^*T *^(τ = 16 h), and *per*^*L *^(τ = 29 h) flies was significantly reduced compared to the wild type flies even under short and long LD cycles [85], thus contradicting the tenets of circadian resonance hypothesis. The reproductive output in many organisms bears an inverse relationship with lifespan. Inferences on fitness advantage based upon lifespan data alone could therefore be misleading, and hence multiple fitness components should be taken into account to assess adaptive significance of circadian clocks [92-96]. The most convincing and perhaps the only unequivocal demonstration of circadian resonance came from extensive and elegant studies on cyanobacteria *Synechococcus elongatus *[6]. In this study the growth rates of various strains of cyanobacteria having different circadian periodicities were assayed while competing against each other. Under pure culture conditions in LL, all strains showed similar growth rates. The wild type (τ = 25 h) and two strains having mutations in the *KaiC *gene (τ = 23 h and τ = 30 h) were competed against each other in various combinations. When two strains were mixed in approximately equal proportions and cultured under LD cycles of 11:11 h and 15:15 h, strains whose clock period matched closely that of the LD cycle always out-competed strains whose clock periods were far removed from those of the LD cycles [6] (Figure 6). These results were reexamined in cyanobacterial strains having mutations on any of the three *Kai *genes (*Kai A*, *KaiB *and *KaiC*). The mutant strains displayed circadian periods ranging between 22 h to 30 h, and in competition experiments, strains whose periodicity matched those of the LD cycles out-competed others whose periods were far removed. Thus, fitness advantages conferred to cyanobacteria via circadian resonance appear to be independent of the genotype but depend upon clock period alone [97]. On the other hand, when mutant strains with dampened bioluminescence rhythm (CLAb) or those showing arrhythmic bioluminescence (CLAc) were competed against the wild type strain under LD 12:12 h regime in mixed cultures, the CLAb and CLAc strains lost to wild-type strains, but out-competed them under LL regime [97] (Figure 7), suggesting that circadian clocks may not be beneficial under all environments and, in fact, may even be deleterious under constant conditions. The authors argued that rhythmic suppression of photosynthesis under LL in the wild type strain probably makes them maladaptive compared to the arrhythmic strains that can photosynthesize continuously under LL. It is quite unlikely that rhythmic photosynthesis in the wild type strain could be maladaptive under LL, as continuous photosynthesis in arrhythmic strains may adversely affect oxygen labile nitrogen-fixation reaction making them no better than the rhythmic strains. As we have seen from the above studies, the results on adaptive significance of circadian rhythm accrued via circadian resonance are mostly conflicting and suggestive, but occasionally conclusive.

## Clocks in the dark

An obvious corollary of circadian resonance hypothesis is that circadian clocks would be of less advantage to organisms living in constant environments such as depth of oceans, underground caves and rivers, or any such aperiodic environments [2]. Therefore, it was believed that organisms living in such seemingly timeless environments would lose the ability to measure time on a circadian scale, and the ability to entrain to periodic environmental cycles. On the contrary, circadian rhythms were found to persist in cave-dwelling fishes [98], in cave-dwelling millipedes [99], and in populations of *D. melanogaster *that had been reared for more than 600 generations under constant laboratory conditions [100-102]. Furthermore, in one of our recent studies we found that eclosion [103] and locomotor activity (Paranjpe et al., unpublished data) rhythms of these flies entrain to a wide range of periodic LD cycles ranging from 20 h to 28 h. In addition, these flies responded to brief light pulses by shifting the phase of their locomotor activity rhythm in a phase-dependent manner, quite similar to the wild type flies maintained under LD cycles (Paranjpe et al., unpublished data). Thus, it appears that important clock features such as period, precision, phase-relationship; phase response properties and ability to entrain to a wide range of LD cycles remain intact in organisms living in constant environments. In absence of cycling environments, where there is no apparent need to synchronize behavioral and metabolic processes with the environmental cycles, persistence of functional clocks and photo-entrainment mechanisms suggests that circadian clocks confer some "intrinsic adaptive advantage" to their owners. The intrinsic advantage of having circadian clocks is probably accrued by facilitating coordination of various internal metabolic processes within the organism [2,84]. The main focus of studies on adaptive significance of circadian rhythms so far has been to investigate extrinsic advantages of possessing circadian clocks in periodic environments, while studies on intrinsic adaptive advantages have always occupied the back seat.

## Concluding remarks

Regulation of behavioural and metabolic processes on a circadian scale has traditionally been thought to be a characteristic feature of eukaryotic organization until it was demonstrated that even prokaryotes such as cyanobacteria possess circadian timing devices. Analysis of sequence data of a large number of prokaryotic genomes revealed that prokaryotic circadian clocks evolved in parallel with the geophysical history of our planet. It is believed that natural selection, multiple lateral transfers, and gene duplications and losses were the major forces that shaped the evolution of early circadian clocks. Besides the periodic biotic and abiotic forces of geophysical environment, the need to segregate metabolic processes according to optimal phases of the environmental cycles also appears to have acted as a force of natural selection that shaped circadian clocks. Irrespective of the disagreements about the forces of natural selection that acted on early clocks, there is a general agreement among circadian biologists that circadian clocks, as they exist now, may have evolved as a tool primarily adaptive to daily cycles of the natural environment. Initially several geophysical cycles may have played crucial roles in exerting selection pressure, while later, daily and seasonal changes may have further fine-tuned them.

Most studies on adaptive significance of circadian rhythms suffer from a number of drawbacks such as the lack of population-level replication, true controls and of adequate control on the genetic composition of the populations, which in many ways limit the potential insights gained from such studies. Moreover, these studies were carried out on mutant and often highly inbred animals. Besides the fact that mutants and inbred lines are likely to yield spurious genetic correlations between fitness components [104] due to genetic drift, it is hard to imagine how evolution of circadian timing systems could have taken place in terms of large changes in one particular gene. On the other hand if we assume that many genes make small contributions to circadian phenotype, then it is far more likely that such genes will be involved in the evolutionary fine-tuning of circadian clocks. Indeed, a number of quantitative trait loci (QTLs) have been identified on the mouse chromosomes 1, 6, 9, 11, 17 and 19 that can potentially contribute to the determination of period of wheel running rhythm in laboratory mice [105]. Variation in the period, phase and amplitude of 150 *Arabidopsis *accessions has also been attributed to QTLs in ARABIDOPSIS PSEUDO-RESPONSE REGULATOR family [106]. Such latitudinal clines in period length, phase and amplitude have been taken as evidence for adaptive significance of circadian timing system [106]. Most studies aimed at resolving issues related to the adaptive significance of circadian rhythms used lifespan as the sole indicator of fitness, though it is well known that higher reproductive output often results in early death. This suggests that one should simultaneously use multiple components of fitness to assess adaptive advantage. Finally, most studies used replication at the level of individual rather than populations, while the unit of replication in any study addressing evolutionary questions needs to be populations, not individuals. Therefore, the overwhelming impression one gets from the studies on adaptive significance of circadian rhythms is one of suggestive, but only occasionally conclusive, results. Perhaps, rigorously designed laboratory selection studies under different environmental conditions might help us examine adaptation as it occurs and the development of circadian organization associated with such adaptation.

## Competing interests

The author(s) declare that they have no competing interests.

## Authors' contributions

DAP and VKS contributed equally to this article.
